# The effect of super brain yoga on the cognitive function of hemodialysis patients

**DOI:** 10.1016/j.heliyon.2024.e36384

**Published:** 2024-08-15

**Authors:** Mahdi Babakhani, Kobra Rahzani, Davood Hekmatpou, Vida Sheykh

**Affiliations:** a School of Nursing, Arak University of Medical Sciences, Arak, Iran; b School of Nursing, Department of Nursing, Arak University of Medical Sciences, Arak, Iran; c School of Nursing, Arak University of Medical Science, Arak, Iran; d School of Medicine, Department of Medicine, Hamadan University of Medical Sciences, Hamadan, Iran

**Keywords:** Cognitive function, Cognitive dysfunction, Hemodialysis, Neuropsychological tests, Super brain yoga

## Abstract

**Introduction:**

Cognitive impairment is one of the most important end-stage consequences of renal disease. This study was conducted to investigate the effect of super brain yoga on the cognitive function of hemodialysis (HD) patients.

**Methods:**

This randomized clinical trial was conducted on 60 HD patients who were assigned to the control (n = 30) and intervention (n = 30) groups. In addition to undergoing their routine HD, subjects in the intervention group performed yoga exercises for one month, at least three days a week, once a day. Cognitive function score of the patients at baseline and after the study (one month later) was measured using the Mini-Mental State Examination (MMSE). Data were analyzed using SPSS version 20 using descriptive statistics, including mean and standard deviation, and inferential statistics, including independent *t*-test, paired *t*-test, and ANCOVA.

**Results:**

The mean score of cognitive function, urea, creatinine, and dialysis adequacy at baseline was 26.07 ± 3.72, 133.83 ± 34.19, 9.37 ± 2.55, and 1.22 ± 0.24 in the control group and28.97 ± 1.62, 174.17 ± 52.8, 13.38 ± 4.16, and 1.26 ± 0.22, in the intervention group, respectively. At the baseline, there was a significant difference between the two groups in terms of cognitive function, urea, creatinine (p-value = 0.001), but there was not in terms of dialysis adequacy (p-value = 0.974). Therefore, the analysis of covariance (ANCOVA) was used to adjust their effects. The mean score of these variables after the study was 25.77 ± 3.11, 146 ± 42.03, 9.7 ± 2.61, and 1.24 ± 0.24 in the control group and 29.17 ± 1.23, 156.03 ± 37.67, 12.27 ± 3.46, and 1.43 ± 0.19 in the intervention group, respectively. There was a significant difference in cognitive function between two the groups (p = 0.05).

**Conclusion:**

Super brain yoga exercises seem to play an effective role in improving the cognitive function of HD patients. Therefore, super brain yoga is recommended as a complementary therapy for HD patients in nursing.

## 1 Introduction

End-stage renal disease (ESRD), also known as chronic kidney disease (CKD) stage 5, is characterized by the patient's need for maintenance dialysis to survive. When kidney function drops below 10–15 % of normal, dialysis or transplantation becomes necessary for survival [1,2]. Hemodialysis (HD) is the most prevalent type of kidney replacement therapy (KRT). Cognition refers to the mental processes involved in knowledge acquisition through reasoning or perception, underlying all activities of daily living from the simplest to the most complex. Cognitive impairment, or intellectual disability, involves decreased mental alertness, intellectual impairment, reduced attention and concentration, memory deficits, and impaired perceptual-motor coordination. This issue has drawn increased attention from healthcare teams in recent years [[3], [4], [5], [6]]. Patients with CKD commonly suffer from some degree of cognitive impairment. Additionally, kidney dysfunction is linked to a more rapid decline in mental function in CKD patients compared to age-matched controls [3]. Depending on the cognitive impairment assessment method and CKD stage, the prevalence of cognitive impairment in CKD patients ranges from 10 to 40 %, with the highest prevalence observed in ESRD patients requiring dialysis [6]. Despite the high risk of cognitive impairment associated with CKD, it often receives insufficient attention in clinical settings [4]. Cognitive impairment diminishes the ability to make informed decisions and comply with dialysis activities and is considered an independent risk factor for mortality in HD patients. Therefore, identifying mild cognitive disorders is crucial for primary prevention and delaying cognitive decline in HD patients [7]. Delirium, characterized by transient organic mental disturbance with marked abnormalities in attention and impaired global cognitive functions, contrasts with dementia, a progressive syndrome of acquired global intellectual impairment occurring in a clear consciousness [8]. Mild cognitive impairment (MCI) or minor neurocognitive disorder is defined by concerns over changes in cognition or impairment in cognitive domains, while functional abilities and independence are preserved and there is no obvious dementia [9]. Uremic toxins and prolonged dialysis duration impair central nervous system function in dialysis patients. CKD patients may develop encephalopathy due to uremic or dialysis-related factors. Additionally, cerebrovascular disease, dyslipidemia, hypertension, oxidative stress, anemia, inflammation, and hyperglycemia in CKD patients increase the risk of cognitive impairment [6,10]. Greater cognitive function impairment is observed in more advanced stages of kidney disease, making early management of cognitive deficits essential [11]. Diagnosing cognitive impairment is important due to its association with increased mortality risk and reduced quality of life in dialysis patients [12]. Cognition is crucial in CKD management as healthy cognition is necessary for self-care in dialysis. Patient participation, choice, and patient-centered decision-making result in better outcomes and are considered best clinical practices. However, there is limited understanding of the cognitive context in which patients make such decisions [11]. Cognitive impairment also increases the time staff spend caring for patients, the use of healthcare resources, and the number and duration of hospitalizations [13]. Pharmacological interventions for cognitive impairments often have side effects such as dizziness, nausea, diarrhea, muscle aches, neutropenia, insomnia, anorexia, headache, and constipation [14]. Consequently, complementary medicine has been considered by researchers [15]. Yoga, a branch of complementary medicine with roots in traditional Indian philosophy, aims to unite mind, body, and spirit through physical postures, breathing exercises, and recommendations for a moral lifestyle and spiritual practice [16,17]. Yoga benefits physical and mental health by increasing body resistance, balance, flexibility, relaxation, and mindfulness, meditation, and the combination of proprioceptive and interoceptive awareness [18]. Super brain yoga, introduced by Master Choa Kok Sui, is noted for its efficiency and simplicity in increasing brain power. This technique reportedly combats mental consequences of aging, memory loss, Alzheimer's disease, and dementia by enhancing mental function. Super brain yoga combines hatha yoga, pranayama, and reflective therapy, channeling energy upward from lower energy centers to the brain, facilitating thinking and concentration, and increasing alpha waves in the brain for relaxation. It harmonizes the brain's hemispheres and integrates brain function without reported side effects [[19], [20], [21], [22], [23], [24], [25], [26]]. Given the side effects of drugs for cognitive disorders and the preventive benefits of complementary therapies, super brain yoga could potentially reduce cognitive problems in HD patients. This study investigates the effect of super brain yoga on the cognitive function of HD patients.

## 2 Materials and methods

**Fig. 1:**
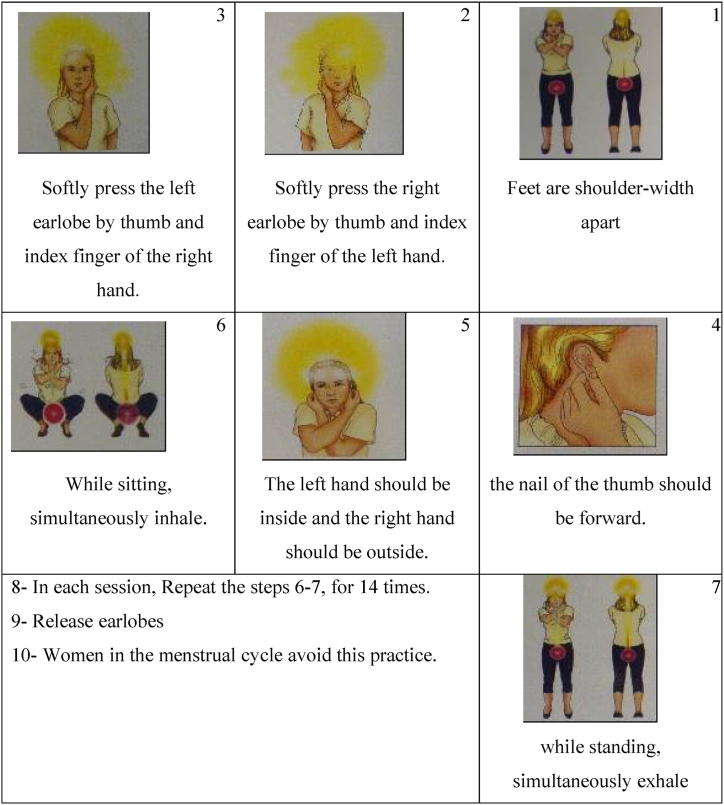
Procedure of Super Brain Yoga [ from: Farahani PV, Hekmatpou D, Khonsari AH, Gholami M. Effectiveness of super brain yoga for children with hyperactivity disorder. Perspect Psychiatr Care. 2019 Apr; 55(2):140–146. https://doi.org/10.1111/ppc.12266. Epub 2018 Feb 10. PMID: 29427513.].

This randomized clinical trial, was conducted on HD patients at Shahid Beheshti Hospital in Hamadan. The required sample size was calculated based on the study of Tayibi et al. [27], considering α = 0.10 and β = 0.20 and 10 % sample loss, which was estimated to be 30 people for each group. The sampling process included targeted sampling based on the inclusion criteria, which was carried out from March 2016 to April 2017. Inclusion criteria were consent to participate, age 18–60 years, at least three months of HD experience, a score above 17 on the Mini Mental State Examination (MMSE), absence of orthopedic or arthritis issues, ability to perform yoga movements, no acute cardiovascular disease, no known mental illness, no drug or psychotropic substance abuse, and full consciousness. Exclusion criteria included changes in clinical symptoms preventing exercise continuation, withdrawal from regular exercise, cessation of HD due to kidney transplantation or other reasons, and performing exercise less than three times a week. All patients underwent hemodialysis three times a week. After project approval and obtaining informed consent, the study was conducted by the researcher and two trained nurses in the HD ward who administered the MMSE questionnaire. Subjects were randomly assigned to control and yoga groups using the block method. In addition to routine HD, the yoga group performed yoga exercises involving deep inhalation and exhalation for one month, at least three days a week, once a day. Super brain yoga was performed as follows: the patient faced east and connected their tongue to the palate. The right earlobe was slowly squeezed with the left thumb and index finger, and the left earlobe was squeezed with the right thumb and index finger, with the left arm inside and the right arm outside. While sitting down, the patient inhaled simultaneously; while standing up, the patient exhaled simultaneously (Fig. 1) [23]. This sequence was repeated 14 times per session [21]. To prevent confounding effects, subjects were asked to perform yoga exercises 12–24 h after the end of the HD session and the next day. The researcher trained the intervention group in super brain yoga and provided photos and videos demonstrating the exercises.

Participants practiced yoga in the presence of the researcher to ensure correct technique and maintained a checklist to record the day and time of exercises. The control group only underwent routine HD. If individuals in both groups were receiving pharmacological therapy for cognitive impairment, their therapy continued and was recorded in a demographic information questionnaire.

Demographic information (age, sex, marital status, occupation, history of mental illnesses, smoking, and drug use) was collected at baseline. The MMSE questionnaire was administered at baseline and post-intervention by two nurses, 12–24 h after the last HD session. Accumulation of metabolic waste products, such as urea and creatinine, and dialysis adequacy, which can affect cognitive ability, were assessed as confounding factors one day before and one day after the study.

Two intervention group members were excluded due to musculoskeletal problems and replaced by two eligible individuals who performed the yoga exercises. Sixty participants completed the study. Cognitive function was assessed using the MMSE at baseline and after the intervention. Data were analyzed using SPSS version 20, employing descriptive statistics (mean and standard deviation) and inferential statistics (independent *t*-test, paired *t*-test, and ANCOVA).

The MMSE is the most widely used screening tool for cognitive impairment [28]. Designed in 1975 by Folstein et al. [29], the MMSE consists of 11 items divided into two parts: verbal answers to orientation, memory, and attention questions, and tasks assessing naming, executing spoken or written commands, writing sentences, and copying shapes. The maximum score is 30, with a cutoff point of 23–24. Scores between 24 and 30 indicate normal cognitive status, 18–23 indicate mild cognitive impairment, and below 17 indicate severe cognitive impairment [30,31].

Marshall et al. validated the MMSE in a study of 206 patients with mental disorders and 63 healthy individuals, finding significant correlations with the Wechsler IQ Scale for adults (0.77 for the oral part and 0.66 for the practical part, both significant at p < 0.0001). Test-retest reliability after 24 h showed a correlation coefficient of 0.88 [32]. Mystakidou et al. found a Cronbach's alpha of 0.89 in 103 cancer patients [33], while a study in Spain reported an alpha coefficient of 0.94 [34]. Seyedian et al. validated the Persian MMSE, with a Cronbach's alpha of 0.81, a cutoff point of 22, and sensitivity and specificity of 90 % and 93.5 %, respectively [35]. This indicates the Persian MMSE's validity for assessing cognitive function.

## 3 Results

According to Fisher's exact test, both groups were homogeneous at baseline in terms of gender, marital status, and smoking status (p > 0.05) (Table 1). However, an independent *t*-test indicated that the two groups were not homogeneous in terms of age, urea levels, creatinine levels, and cognitive function status (p < 0.05) (Table 2). Therefore, analysis of covariance (ANCOVA) was employed to adjust for these differences post-intervention. At baseline, the mean scores for cognitive function, urea levels, creatinine levels, and dialysis adequacy were 26.07, 133.83, 9.37, and 1.22 in the control group, and 28.97, 174.17, 13.38, and 1.26 in the intervention group, respectively. After the study, the mean scores were 25.77, 146, 9.7, and 1.24 in the control group, and 29.17, 156.03, 12.27, and 1.43 in the intervention group, respectively (Table 2, Table 3, Table 4, Table 5). Therefore, there was a significant difference between the intervention and control groups in improving cognitive function after the yoga exercises (p = 0.05).

**Table 1:** Demographic characteristics of the control and yoga groups.

Variables Categorical		Control group		Yoga group		P-value
		N	%	N	%	
**Gender**	Male	14	46.7	19	63.3	0.194
	Female	16	53.3	11	39.7	
	Total	30	100	30	100	
**Marital status**	Single	5	16.7	4	13.3	0.718
	Married	25	83.3	26	86.7	
	Total	30	100	30	100	
**Smoking Continuous**	Yes	2	6.7	3	10	1.00
	No	28	93.3	27	90	
	Total	30	100	30	100	
**Age**		Mean	±SD	Mean	±SD	0.004
		51.07	10.11	42.97	10.82

**Table 2:** Mean score of cognitive function in the studied groups before and after the test.

Group	**Pre-test**	**Post-test**	**Marginal mean ± SD**
	Mean ± **SD**	Mean ± **SD**	
Control	26.07 ± 3.72	25.77 ± 3.11	25.99 ± 3.67
Yoga	28.97 ± 1.62	29.17 ± 1.23	29.07 ± 1041
P – value	<0.001^a^	0.001^b^	

a Independent *t*-test.

b Adjusted for age, urea levels, creatinine levels, and pre-study cognitive function (ANCOVA).

**Table 3:** Mean urea score levels in the studied groups before and after the test before and after the study.

Group	**Pre-test**	**Post-test**	**Marginal mean ± SD**
	Mean ± **SD**	Mean ± **SD**	
Control	133.83 ± 34.19	146 ± 42.03	139.91 ± 35.77
Yoga	174.17 ± 52.80	156.03 ± 37.67	165.1 ± 40.21
P – value	0.001^a^	0.438^b^	

a Independent *t*-test.

b Adjusted for age, urea levels, creatinine levels, and pre-study cognitive function (ANCOVA).

**Table 4:** Mean score of creatinine levels in the studied groups before and after the test.

Group	Pre-test	Post-test	Marginal mean ± SD
	Mean ± SD	Mean ± SD	
Control	9.37 ± 2.55	9.70 ± 2.61	9.53 ± 3.45
Yoga	13.38 ± 4.16	12.27 ± 3.46	12.83 ± 3.44
P – value	0.001^a^	0.003^b^	

a Independent *t*-test.

b Adjusted for age, urea levels, creatinine levels, and pre-study cognitive function (ANCOVA).

**Table 5:** Mean score of dialysis adequacy in the studied groups before and after the test.

Group	**Pre-test**	**Post-test**	**Marginal mean** ± **SD**
	Mean ± **SD**	Mean ± **SD**	
Control	1.22 ± 0.24	1.24 ± 0.24	1.23 ± 0.23
Yoga	1.26 ± 0.22	1.43 ± 0.19	1.35 ± 0.19
P – value	0.471^a^	0.974^b^	

a Independent *t*-test.

b Adjusted for age, urea levels, creatinine levels, and pre-study cognitive function (ANCOVA).

## 4 Discussion

The findings of this research revealed a significant improvement in cognitive function in the yoga group compared to the control group. As far as the authors know, no prior studies have specifically examined the effect of yoga on the cognitive function of hemodialysis (HD) patients; however, other studies have evaluated the effect of yoga on cognitive function in patients with different conditions. These studies generally show that yoga improves cognitive function. For example, Hekmat Pou et al. demonstrated that super brain yoga exercises reduce the symptoms of Autism Spectrum Disorder (ASD), a neurodevelopmental and neuro-cerebral disorder in children. In that study, the mean severity of autism significantly decreased post-intervention in the intervention group (p ≤ 0.001), indicating an improvement in the individual's status. Similarly, in our study, the mean score of cognitive function increased post-intervention in the intervention group (p < 0.001), indicating an improvement in patients' cognitive status. Both studies used super brain yoga over similar time periods and targeted cognitive function, which is one of the higher brain functions. This suggests that super brain yoga might improve cognitive status through mechanisms that promote brain integration and balance. Lin et al. (2015) conducted a randomized controlled trial comparing the effects of aerobic exercise and yoga on improving neuro-cognitive function in women with psychosis. They found cognitive function improvement in both the yoga and aerobic exercise groups compared to the control group, particularly in verbal acquisition, memory, and attention in the yoga group. They suggested that yoga enhances mental focus, body control, and brain structure and function, leading to cognitive improvements [36]. Our study similarly found significant improvements in memory and attention in the yoga group, indicating that different types of yoga can enhance cognitive function in various patient populations. Talwadkar et al. (2014) showed that trataka yoga improved cognitive functions, including short-term memory, working memory, attention, concentration, visual scanning, and executive function in the elderly. Their intervention lasted 26 days and included exercises like eye movements, candle gazing, breathing, and chanting. This suggests that yoga practices involving focused attention and relaxation can enhance cognitive function [37]. In a study by Saoji et al. (2017) on the mind sound resonance technique (MSRT), they found that a 30-min MSRT session could improve cognitive functions such as attention, concentration, visual scanning, psychomotor speed, mental flexibility, and information processing speed in medical students. They attributed these improvements to reduced anxiety and the deactivation of the limbic system due to chanting [38]. Our study's use of super brain yoga, which involves similar relaxation and mental focus elements, showed improvements in cognitive function, particularly attention.

Gallego et al. conducted a quasi-experimental study on the effect of hata yoga on patients with Alzheimer's disease and found improvements in working memory, attention, and concentration, which they attributed to increased brain blood flow and physical activity [39]. Some studies, however, have reported no effect of yoga on cognitive function. For instance, Oken et al. (2004) found no improvement in cognitive function in patients with multiple sclerosis (MS) after yoga or aerobic exercise [40]. Notably, they focused only on attention, and other cognitive functions might have been affected differently.

Several factors, such as the accumulation of uremic toxins and prolonged dialysis duration, impair the central nervous system function and worsen cognitive impairment in HD patients. Uremic toxins damage brain structures, such as the frontal lobe and basal ganglia, which have been detected in brain imaging studies of these patients. Super brain yoga increases alpha waves in the brain, promoting relaxation, harmonizing the brain hemispheres, and enhancing brain integration. It also boosts neurotransmitter formation and release, activates specific neural pathways, and increases post-synaptic membrane sensitivity while inhibiting distracting signals, thereby improving cognitive performance [24].

Cognitive function in HD patients is subject to significant fluctuations due to the rapid removal of uremic toxins during dialysis. This variability, along with differences in dialysis methods and adequacy, complicates the assessment of yoga's effects on cognitive function. Despite these confounding factors, our findings suggest that super brain yoga can be a valuable complementary therapy for cognitive impairment in HD patients.

Given its simplicity and short duration, super brain yoga can be easily integrated into nursing practice, education, and research. Teaching this technique to patients in medical centers could help prevent cognitive disorders and improve cognitive status and quality of life. Nursing students can also utilize this technique as a complementary treatment for patients. This research opens avenues for further studies to explore the broader applications and benefits of super brain yoga.

## 5 Conclusion

The results of our study showed that mind-enhancing yoga is an easy, non-invasive and safe intervention that can significantly improve the cognitive function of HD patients. Mind strengthening yoga is an intervention that does not require special equipment, it is practical and easy to perform, it is accepted by patients, so it is suggested to use the above yoga to improve the cognitive function of HD patients and the method of its implementation should be taught to patients and their health care workers, especially nurses.

## Limitations

The duration of our intervention in this study was one month, it is likely that the intervention in a longer period of time would be associated with greater effects of yoga in the intervention group. On the other hand, we were not able to perform the exercises until 12–24 h after HD due to the fatigue and lethargy of the patient and we were forced to do the exercises every other day instead of every day, therefore, it is suggested to perform this intervention in these patients for a longer period of time and with repeated interventions. Also, we could not perform this type of yoga in HD patients with acute cardiovascular diseases and limited activity and orthopedic problems such as arthritis, it is recommended to conduct a research using corrective yoga.

## Funding statement

This work was supported by the Research Council and the Ethics Committee of 10.13039/501100007113Arak University of Medical Sciences (Ethics code: IR.ARAKMU.REC.1396.254).

## Additional information

No additional information is available for this paper.

## Ethics declarations

This study was reviewed and approved by the Research Council and the Ethics Committee of Arak University of Medical Sciences (Ethics code: IR.ARAKMU.REC.1396.254). It was registered at the Iranian Registry of Clinical Trials (No.: IRCT20180908040965N1). All patients provided informed consent to participate in the study. All patients provided informed consent for the publication of their anonymized case details and images.

## Data availability statement

Data associated with our study hasn't been deposited into a publicly available repository. The datasets used during the current study are available to the research team (authors). Data will be made available on reasonable request.

## CRediT authorship contribution statement

**Mahdi Babakhani:** Writing – review & editing, Writing – original draft, Methodology, Investigation, Formal analysis, Data curation. **Kobra Rahzani:** Writing – review & editing, Writing – original draft, Validation, Supervision, Project administration, Investigation, Formal analysis, Conceptualization. **Davood Hekmatpou:** Writing – original draft, Supervision, Methodology, Formal analysis, Data curation. **Vida Sheykh:** Writing – original draft, Validation, Supervision, Methodology.

## Declaration of competing interest

The authors declare that they have no known competing financial interests or personal relationships that could have appeared to influence the work reported in this paper.
